# Neuroimaging and Neuropsychological Studies in Sports-Related Concussions in Adolescents: Current State and Future Directions

**DOI:** 10.3389/fneur.2019.00538

**Published:** 2019-05-24

**Authors:** Shalini Narayana, Christopher Charles, Kassondra Collins, Jack W. Tsao, Ansley Grimes Stanfill, Brandon Baughman

**Affiliations:** ^1^Department of Pediatrics, University of Tennessee Health Science Center, Memphis, TN, United States; ^2^Department of Anatomy and Neurobiology, University of Tennessee Health Science Center, Memphis, TN, United States; ^3^Le Bonheur Children's Hospital, The Neuroscience Institute, Memphis, TN, United States; ^4^Department of Neurology, University of Tennessee Health Science Center, Memphis, TN, United States; ^5^Le Bonheur Children's Foundation Research Institute, Memphis, TN, United States; ^6^Department of Neurology, Memphis Veterans Affairs Medical Center, Memphis, TN, United States; ^7^Department of Acute and Tertiary Care, University of Tennessee Health Science Center, Memphis, TN, United States; ^8^Semmes Murphey Neurologic and Spine Institute, Memphis, TN, United States; ^9^Department of Neurosurgery, University of Tennessee Health Science Center, Memphis, TN, United States

**Keywords:** mild traumatic brain injury, neuroimaging, neuropsychological testing, accelerometers, fMRI, DTI, MEG, TMS

## Abstract

Sports-related concussion, is a serious neurological concern that many adolescent athletes will face during their athletic careers. In some instances, the effects of sports-related head injury are long-lasting. Due to their still-developing brains, adolescents appear to be more vulnerable to long-term repercussions of these injuries. As all sports-related concussions are mild traumatic brain injuries (mTBI), this review we will examine the pathophysiology of mTBI, its acute effects and long-term risks from sustaining injury, and current and needed advancements in the areas of neuropsychological testing, accelerometer telemetry, and neuroimaging. Current methods do not adequately measure the extent of an injury that an athlete may sustain, potentially putting these athletes at a much greater risk for long-term effects. To better understand mTBI, neuropsychological testing best practices need to be developed, standardized, and implemented based on sound scientific evidence in order to be propagated as clinical guidelines. Wearable accelerometers can be used to assess thresholds for mTBI and cumulative effects of concussive and subconcussive injuries. Novel neuroimaging methods that can detect anatomical abnormalities and functional deficits with more specificity and sensitivity should be developed. Young athletes are particularly a vulnerable population warranting immediate and significant research aimed at protecting them against sports related injury and mitigating their long-term deficits.

## Introduction

Sports-related concussion (a type of mTBI) is a serious neurological concern that many adolescent athletes will face during their athletic careers. Sports-related concussion is the second most common cause of head injury in children, second only to motor vehicle accidents (1). It has been estimated that 1.6 to 3.8 million concussions occur in sports and recreational activities each year in the United States (1). The highest incidence for sports-related concussion in young male athletes is reported in high school football, whereas soccer and basketball show the highest prevalence in female athletes (2). Emerging research suggests that younger athletes take longer to recover and may be more vulnerable than adults to the effects of an mTBI (3). Thus, the problem of mTBI in youth or adolescents needs urgent and extensive investigation, especially regarding diagnosis, management, and return-to-play guidelines. Mismanagement of mTBI puts athletes at risk for the short-term risk of second impact syndrome as well as long-term neurological sequelae such as chronic traumatic encephalopathy and chronic neurocognitive impairment (4). In this article, we will review the current understanding of the pathophysiology of mTBI, and highlight neuropsychological testing and neuroimaging methods that can be used to detect and monitor recovery of a youth sustaining an mTBI.

### Brain Development

Prior to discussing the potential effects of mTBI on adolescents, it is important to understand normal brain development in this population. Although a child's total brain volume reaches ~90% of its adult size by 5 years of age, the brain continues to undergo significant changes throughout childhood and adolescence (5). Studies have demonstrated an increasing volume of white matter and reorganization of synaptic connections throughout the adolescent period (6–8). Gray matter in frontal, parietal, and temporal lobes reach their maximum volume around 12–16 years of age, while the gray matter in the occipital lobe continues to increase in size and density until 21 years of age (9). While gray and white matter changes in association cortices continue through the second decade of life, the greatest delay in maturation occurs in the prefrontal cortex, where myelination and synaptic pruning continues well into adulthood (10, 11). Similarly, the posterior part of corpus callosum continues to increase during adolescence as a result of increased myelination in the interhemispheric white matter tracts (12). These processes can be altered by environmental factors, including head injury.

Thus, it is reasonable to hypothesize that young children involved in contact sports could have a more prolonged course of recovery following mTBI due to the physiologic immaturity of their brains as compared to adults. In fact, recovery times in children average approximately 1 month while symptoms in adults generally resolve within 10 days (3). Additionally, studies of adolescents have shown impaired working memory up to 1 year from injury and impaired attention up to 2 years from injury (13). These findings suggest that children are at risk for permanent impairments following mTBI (13). In addition, children with a prior history of migraines, learning disabilities, and attention deficit disorder may experience an even further prolonged course of recovery as well as more severe symptoms (4). Furthermore, injuries sustained during childhood and adolescence has the potential to change the trajectory of brain development, resulting in long lasting effects well into adulthood.

### Definition of mTBI and Its Effects

An mTBI can be defined as a temporary alteration in brain function and mental status resulting from blunt trauma to the head or body that rapidly displaces the brain within the skull (14). It is important to note two items surrounding this definition (1) all concussions are mTBI, but not all mTBI are concussions; and (2) In any blunt trauma to the head or body, load of that force is also transmitted and causes fracture, contusion, hemorrhages, which are not addressed in this review of milder injuries. A loss of consciousness can occur, but is not required for a head injury to be classified as an mTBI (14). Typically, mTBI is characterized by rapid onset of symptoms that last for a short time and resolve spontaneously. Symptoms may develop more slowly in some individuals, so athletes suspected of suffering an mTBI should be assessed multiple times following an impact (15). Headache and dizziness are the most common physical symptoms of mTBI (16). Other physical symptoms include fatigue, photosensitivity, and nausea (15). Patients with mTBI also report cognitive symptoms such as an inability to concentrate (“feeling foggy”), as well as various other problems with memory and confusion (15). Emotional symptoms can include anxiety, irritability, emotional lability, and depression (4). Patients with mTBI may also experience difficulty falling asleep, excessive drowsiness, and/or other changes in sleep habits (4).

While one instance of concussion is not ideal, those young players who sustain multiple concussions over the course of their career may have even bigger cause for concern. A history of a previous concussion is associated with a greater risk of sustaining another concussion and puts athletes at an increased risk for longer-term deficits (16). Young athletes who sustain 3 or more concussions are more likely to exhibit a greater symptom burden including loss of consciousness, post-traumatic amnesia, and confusion (17). Furthermore, on neuropsychological testing, cognitive symptoms are found to persist longer for those with a history of 2 or more concussions (18). In addition, the grade point averages of student athletes with 2 or more concussions have been shown to be significantly lower (18).

Athletes with an history of multiple concussions are more likely to suffer from long-term neurological sequelae including deficits of working memory and visuospatial processing, epilepsy, early-onset Alzheimer's disease, chronic depression, and chronic traumatic encephalopathy (19). Second-impact syndrome can cause severe cerebral vascular congestion and result in death (2, 20). There are currently no long-term prospective studies that examine the effects of concussions in young athletes who participated in contact sports before the college and professional levels. Future studies should be directed at understanding the effects of concussions, and the cumulative effects of repeat concussions throughout the entire career of young athletes.

## Pathophysiology of mTBI

An mTBI can be caused by direct trauma to the head or the rotational acceleration of the brain within the skull. These changes result in cellular damage to neurons, axons, and the vasculature of the brain which disrupts neurotransmission and neurometabolism (21, see 22 for a review). Animal models have provided the majority of information regarding the pathophysiology of the damage done by mTBI (2). These studies have demonstrated that a concussive injury damages the neuronal cell membrane, beginning a cascade of abnormal neuronal events. Disruption of the membrane causes an efflux of potassium to flow back into the extracellular space coupled with influx of sodium and calcium and thereby prompting the release of glutamate, an excitatory amino acid from the neuron (2, 21). Glutamate triggers the release of potassium that in turn causes further depolarization of the cell (2). As the sodium-potassium ion pumps attempt to compensate for this ion imbalance, adenosine triphosphate and glucose stores are depleted. The accompanying decrease in cerebral blood flow then leads to an energy crisis and lactate accumulation (2, 21). In addition to the potassium imbalance, calcium builds up in cell at the same time, thereby impairing mitochondrial function and exacerbating the energy crisis (22). All of these events can also release free radicals and lead to long lasting injury to neurons making them vulnerable to repeat injury (2).

Apart from these acute changes, more protracted impairments in metabolism lasting several days are also noted. Several animal studies have investigated other molecular impacts of mTBI. Across studies, changes in brain amino acids including N-acetylaspartate (NAA), arginine and γ-aminobutyric acid (GABA) and ATP/ADP ratio were noted, illustrating the energy crisis resulting from mTBI (23–26). In addition the degree and duration of the changes in these amino acids correlated with the severity of mTBI (23). These results highlight the role of the initial impact has on the cascade of events that follow, including the changes to metabolism. Further, in animals sustaining multiple mTBIs, the severity of the metabolic alterations increased and, did not demonstrate complete recovery despite prolonged follow up (24). Alterations in glycolytic gene expression and enzymatic activities have been reported following mTBI, indicating to genetic factors mediating brain responses to injury (27). The results of these studies warrant further investigation, particularly to investigate how these results may be applied to the discovery of drugs that could target specific mitochondrial functions and prevent the catastrophic effects of mTBI (26).

In addition to the energy crisis, the biomechanical impact has been shown to damage the cytoskeleton in the form of collapse of neurofilaments and disruption of microtubules. Such injury to axons can lead to impairments in axonal transport, neurotransmission and even axonal disconnection (21). Emerging data also indicate that proteins like integrins that form cytoskeletal anchors in the cell membrane in both neurons and vascular endothelium likely are the main molecular target of mTBI (21). These axonal changes have been shown to correlate with impaired cognition in both animal models and human studies (21). Additional sequalae of mTBI includes disruption of excitatory and inhibitory neurotransmitter balance, activation of inflammatory cytokines, and in extreme cases cell death.

It is important to note that these pathophysiological changes can persist beyond the resolution of clinical symptoms. Since the cellular functions can take several days to weeks to return to baseline, the patients remain physiologically vulnerable during this period (4). Thus, a second injury during this phase may result in a worsening of the original effects and create increased risk for additional long-term sequelae (4). It has also been shown that younger individuals are more susceptible to the cumulative effects of repeat concussions, most likely due to developmental differences such as brain size, myelination levels, and differences in cerebral blood flow (4). Further studies that examine the pathophysiology of such brain trauma in adolescents are needed to better understand the underlying mechanisms of such injury and recovery. This work is critically needed for better diagnosis and for information on when it is safe to return to normal activities.

## Current State of Diagnostic Methods in mTBI

Guidelines for mTBI diagnosis, management, and return-to-play decisions have been released by the American Medical Society (AMS) of Sports Medicine, the American Academy of Neurology (AAN), and the Concussion in Sport Group (CISG). The AMS and AAN guidelines were last updated in 2013, while the CISG more recently updated its consensus statement in 2016. Despite these three highly regarded guidelines, there is no one agreed-upon protocol for when a child should return-to-play. However, they all recommend that athletes exhibiting symptoms of concussion should be seen by a licensed health care professional or neuropsychologist and that return-to-play should be gradual once symptoms both at rest and with exertion are resolved (28).

### Neuropsychological Testing in mTBI

There is a longstanding history of neuropsychological testing in the objective assessment of cognitive and behavioral changes in individuals with mild traumatic brain injury. It is well-established that individuals with mild traumatic brain injury can suffer neurocognitive deficits, most notably in the areas of processing speed/reaction time, attention, working memory, executive function, and memory (retrieval); these domains of function are readily assessed with formal neuropsychological testing. Several case-controlled and meta-analytic reviews are available on this topic, establishing the reliability and validity of testing in this population (29–34). A detailed discussion of these meta-analytic studies is beyond the scope of the current review; however, we would note the general trend of these meta-analyses, which have suggested that neurocognitive effect sizes tend to diminish spontaneously as the post-injury window broadens. In adult samples, a majority of the variance in post-concussive cognitive complaints has been found to be primarily related to psychological distress factors and litigation.

In the context of sports-related concussion management, the seminal work of Dr. Jeffery Barth and the sports laboratory assessment model at the University of Virginia provided a model of baseline and post-injury assessment that has become a model assessment strategy in many contemporary systems (35, 36). This strategy involves measuring cognition in the asymptomatic athlete, then re-assessing cognition (with the same testing battery) in the injured patient. When baseline testing is available, one is able to not only compare scores of the injured patient to the general population (norm-referenced measurement), but also a more sophisticated intra-individual assessment of change (baseline vs. post-injury). Over time, the use of neuropsychological testing has expanded in the assessment and management of concussion, especially in the field of sports medicine. An inter-organizational neuropsychology group (American Academy of Clinical Neuropsychology, American Board of Neuropsychology, Division 40 (Neuropsychology) of the American Psychological Association, and the National Academy of Neuropsychology) produced a consensus statement suggesting neuropsychologists can provide value-added data in the assessment and management of concussed athletes (37).

The Concussion in Sports Group (CISG) is an international, multi-disciplinary clinical and research group which have published sports concussion consensus guidelines since 2001, with the most recent iteration coming from the group's 5th meeting in 2017 (38). The CISG has addressed neuropsychological/cognitive assessment in their statements. Initially, there was a recommendation that formal cognitive assessment should be an essentially universal component of concussion assessment and management. Over time, this recommendation has been tempered. Specifically, in CISG-5, the group asserted that neuropsychological testing still contributes significant information in sports concussion; however, they found limited evidence suggesting neuropsychological testing prevented concussions or altered concussion outcomes. They go on to state that the current literature fails to support mandatory global neuropsychological testing for all athletes, including both baseline and post-injury assessment. Return-to-school planning was considered to be an area where neuropsychological testing can be especially helpful. More recently, the Centers for Disease Control reviewed literature pertaining to the diagnosis and management of concussion in children. Cognitive testing was discussed, with the overall summary suggesting moderate evidence supporting the use of graded symptom checklists in distinguishing injured patients. In contrast, brief cognitive screening (e.g., Standardized Assessment of Concussion), computerized measures (e.g., CNS Vital Signs), and specific measures of motor reaction time and vestibular-ocular motor screening were found to be of low to very low confidence. We would note that the review did not include studies with more detailed or robust neurocognitive batteries that would commonly be use in a typical clinical neuropsychological battery.

Of course, traditional neuropsychological testing in the context of mTBI/concussion evaluation and management is not without limitations. As noted above, the ability to detect neurocognitive impairments drops off within a few days to weeks following injury, and in some cases, performance-based deficits will resolve before subjective symptoms. Thus, if a clinician is reliant on neurocognitive testing data as the primary marker of recovery, they may risk false negative errors, which further risks prolonged recovery or re-injury in a patient who may still be in the midst of neurologic recovery. Another limitation are interpretations of psychometric change scores. For example, confident interpretation relies on adequate test-retest reliability, which for some tests may be poor, include significant practice effects, or simply not be available (39). Although statistical methodologies are available to control for reliability and practice effects (i.e., reliable change indices, regression-based change scores), the statistical knowledge required to calculate these changes scores often requires formal training, which a vast majority of clinicians may not be exposed to. Other limitations are more pragmatic in nature. For example, large-scale baseline testing with traditional neuropsychological tests can be challenging as they require one-on-on administration, with scoring and interpretation of results requiring trained neuropsychologists (40). Of course, this has been shown to be feasible in some settings. For example, the National Hockey League has employed a dual-approach, employing general screening instruments, computerized assessment, and traditional neuropsychological (paper and pencil) testing (41). Of course, this protocol does not easily generalize to a majority of concussion clinics or general medical practices due to the availability of specialists, time, space, and financing.

Given the changing landscape of concussion, including increased awareness of injury and market pressures for rapid diagnosis and assessment, recent trends have seen the development of computer-based neuropsychological testing. Overall, computerized neuropsychological tests (CNTs) is somewhat of a mixed bag with regard to advantages over traditional measures (see Table 1). In a joint position paper by the American Academy of Clinical Neuropsychology and the National Academy of Neuropsychology, the panel agreed that CNTs can be administered by a wide range of professionals and distributed to a wide clinical population; however, caution was encouraged for users potentially viewing these instruments as a “plug and play” strategy that provides a diagnosis or interpretive statement at the exclusion of meaningful factors when considering cognition (e.g., similarities between normative data setting and clinical testing environment; cultural factors; malingering and effort problems; test theory and psychometric development; degree of computer naivete) (43). The authors of the paper also encourage a clear understanding of the difference between *neuropsychological testing* vs. *neuropsychological assessment*, with the latter implying clinical activities (i.e., clinical history integration, behavioral observations, physical findings, and interpretive and diagnostic conceptualization) beyond the relatively simple task of test administration. Considering the advantages and disadvantages of CNTs, the selection of which CNT instrument to use presents its own problem. Limited studies have considered head-to-head comparisons between CNTs, limiting one's ability to optimally select an instrument with the operational characteristics for their population at hand. Numerous studies have considered psychometric properties of common CNTs, utilized in concussion populations, with results suggesting quite a bit of variability across measures and across test-retest intervals (44, 45). Select studies have attempted to address this gap in the extant literature. Resch et al. (45) examined the psychometric properties of four of the most widely employed CNTs, including the Immediate Post-Concussion Assessment and Cognitive Test (ImPACT), Axon/CogSport, Automated Neurocognitive Assessment Metrics (ANAM), and the Concussion Resolution Index (CRI). Resch and colleagues failed to identify superiority with any specific test and found inconsistent psychometrics across instruments, including varying validity, test-retest reliability, sensitivity, and specificity. Even with ImPACT, a measure that has become ubiquitous with sports concussion assessment, demonstrated mixed validity results (*r* = 0.20–0.88), mixed to unacceptable reliability (*r* = 0.23–0.88), and a broad range of sensitivity (79.2–94.6%) and specificity (89.4–97.3%) (44–46). In another similar study, Nelson et al. (47), compared reliability and validity between the ImPACT, ANAM, and Axon Sports/CogState over 24 h and 8, 15, and 45 day intervals. Sensitivities tended to be strongest at short intervals (47.6–67.8%); however, test-retest reliabilities dropped off for all instruments over longer intervals.

**Table 1:** Advantages and disadvantages specific to CNT.

**Advantages**	**Disadvantages**
Easily administered to large groups (37)	Group administration could potentially alter individual results (38)
Increased availability of multiple assessments (37)	Computers introduce the potential for software and hardware failure; many tools require internet access (39)
Portability allows rapid assessment and testing in more remote environments (37)	Most CNT still require scoring and interpretation by trained neuropsychologists (40)
Electronic medium facilitates compilation of a centralized database for normative data and research (37)	Difficulties remain for interpretation in the absence of baseline data or until complete normative data has been established (42)
Improved accuracy of time-based response measurements (37)	
Test forms can be modified to adjust for individualized presentation (37)	

The extant literature has not identified a “perfect” neuropsychological test for individuals who have suffered concussion. As such, many have advocated for a multidimensional test to manage mTBI (such as the SCAT5) that considers observable symptoms as well as cognitive and neurological screening (4, 48, 49). Within these trends, some have recommended that performance-based testing be geared toward and focused on vulnerable populations, such as children and adolescents, when feasible; however, this data in isolation should not be used as a litmus test to determine an athlete's readiness to play (49). We would note that return-to-play decision making has received a vast majority of the attention with regard to clinical decision making. In youth athletics, we would argue that return-to-learn would be a more significant outcome. Unfortunately, there have been limited studies with respect to the value-added nature of neuropsychological testing in school decision making, which we would expect to be much greater. In order to advance the science, future efforts must work toward establishing a best practice guideline for the use, administration, and interpretation of neuropsychological tests, regardless of the modality. Although some have suggested neuropsychological testing can be helpful even in the absence of an asymptomatic baseline exam, further studies are needed to understand what populations may or may not need baseline examinations (50). Moreover, studies should also be geared toward addressing questions regarding standardization of administration practices and hardware/technology used for CNTs, as well as ongoing studies to establish psychometric and operational characteristics of various instruments, across diverse age, sex, sport, intellectual and educational levels, in order to optimize measure selection (40, 51). The establishment of these guidelines would also be best served with a clearer understanding of the relationship between variations in an individual's scores and the recovery curve for each individual assessment (45).

### Accelerometer Telemetry

The biomechanics of head injury can be assessed using wearable accelerometers that precisely measure the force of impact and provide real-time assessments of linear and angular acceleration as well as g-force. Being unobtrusive, these accelerometers can be worn discretely behind the ear or within headbands and caps and allows monitoring of both helmeted and non-helmeted athletes. Since these devices transmit data in real-time, qualified professionals can be alerted in case of high impact events. While one study using accelerometers embedded in helmets in collegiate football players found no correlation between acute symptoms of mTBI, postural stability, and neuropsychological deficits and the impact magnitude or impact location (52), a more recent study found that the athletes suffering concussion who demonstrated persistent neurophysiological deficits after despite complete symptom resolution had received significantly higher number of side impacts (53). These findings indicate that number and type of impact may be just as important as the force of impact in determining the immediate and long-term sequelae following mTBI.

### Neuroimaging in mTBI

Current standards of care for examining an mTBI include neuroimaging methods such as computed tomography (CT) scans and magnetic resonance imaging (MRI). A CT scan is usually performed first to rule out intracranial hemorrhage and skull fracture (2). While useful in this context, artifacts caused by beam hardening and partial volume effects limits the visualization of posterior fossa, frontal, and temporal regions. Further, CT cannot effectively resolve the gray-white matter interface for mTBI, resulting in poor visualization of deep white matter tracts, hippocampus and brainstem-structures that can be better visualized by MRI (54). However, structural MRI is still not sensitive enough to identify changes in the microstructure of the white matter. Therefore, neither CT nor MRI has the sensitivity or specificity of identifying mTBI related brain injury. Furthermore, it is important not only to determine the specific structural changes, but also functional consequences that occur as a result of an mTBI. We will discuss the current state of neuroimaging methods in turn.

### Structural Imaging-Diffusion Tensor Imaging (DTI)

Diffusion tensor imaging (DTI) is superior to structural MRI and CT scans in detecting white matter alterations, localized lesions, and diffuse axonal damage, especially in younger children where such alterations are typically more difficult to detect (2, 55). DTI provides information regarding white matter microstructure and fiber tract integrity by measuring the motion of the water molecules within the brain. Changes in fractional anisotropy (a measure of the direction of water movement within axons) and mean diffusivity (a measure of the overall diffusion in a tissue) are able to indicate alterations in the structure of white matter after mTBI (3, 56–58). White matter abnormalities have been found in the anterior corona radiata, the uncinate fasciculus, corpus callosum, inferior longitudinal fasciculus, and the cingulum bundle of patients that exhibit persistent cognitive impairment after mTBI (55). Changes in attention and memory have also been associated with altered fractional anisotropy levels within the left anterior corona radiata and uncinate fasciculus (59).

The global changes in fractional anisotropy and radial diffusivity have been seen even months after the initial concussive injury (56). Many of these fractional anisotropy changes are localized to the temporo-occipital white matter (60), but deep white matter changes may show an even longer time course of recovery, especially for female athletes. In a study of female contact sport athletes, microstructural changes could be persistently identified, even as long as 7 months after the initial injury occurred, and despite these athletes denying the presence of post-concussive symptoms (61). These data highlight the importance of improving our current diagnostic methods to better understand structural changes related to mTBI.

### Magnetic Resonance Spectroscopy (MRS)

Magnetic resonance spectroscopy measures the level of metabolites in the brain non-invasively by quantifying the changes in the proton spectrum induced by the metabolites. Individual metabolites and their concentrations can be quantified by their characteristic pattern of resonance frequencies. While MRS can detect signals from many metabolites, it is limited primarily by the concentrations of these metabolites in the tissue being examined and the magnetic field strength of the MRI scanner. Most common metabolites studied using MRS include N-acetylaspartate (NAA) and creatine (indices of neuronal viability), choline (measure of cell membrane integrity), glutamate and glutamine (assess excitatory neurotransmission), and γ-aminobutyric acid (GABA, an inhibitory neurotransmitter). The main advantage of MRS is its ability to monitor alterations in metabolic function over time even in the absence of structural abnormalities.

Researchers are beginning to use MRS to examine the association between mTBI and mitochondrial metabolism. Acutely decreased levels of NAA were noted in athletes who had suffered a concussion (< 3days), similar to that observed in rodent studies. The levels of NAA returned to non-concussive levels by 30 days (62). Additional alterations in concentrations of Creatine and Choline in the frontal lobe white matter have been observed that progressively return to normal by 30–45 days post injury (63). These findings highlight the possible clinical application of MRS as a non-invasive method of assessing changes caused by mTBI that are not evident by routine clinical, neuropsychological, or imaging evaluations (62).

However, MRS does not have the spatial resolution of an anatomical MRI and provides information on metabolites in small areas of the brain. Usually, in each scanning session, one or two areas of interest as well as control regions can be studied, making a priori identification of the critical brain areas a necessity. For example, only few brain areas including the frontal white matter, primary motor cortex, cingulate cortex and thalamus have been studied using MRS (64). But, due to differences in types of sports and locations of impact, there is no clear consensus on which brain areas should be examined. This problem is further compounded by on-going developmental changes in brain metabolites in adolescent athletes as well as normal variations of metabolite concentrations across different brain areas and individuals. Recent advances in whole brain MRS imaging will be helpful in studying metabolic consequences of sports related concussion particularly in children.

### Functional Imaging-Functional Magnetic Resonance Imaging (fMRI)

Functional MRI (fMRI) measures brain activity by detecting changes in blood flow in real time as an individual is presented with a task (2, 65). Through such functional imaging assessments it is possible to associate any alterations in this blood flow with neurocognitive dysfunction post injury. The tasks that are presented during fMRI can be personalized for detection of deficits for each individual concussive case, and thus provide the most specific and accurate information for each patient (65). Another advantage of fMRIs is the capability to run multiple cognitive tests and trials within a short period of time (65). Such multicomponent studies could potentially elucidate the effect of concussive events on the brain, and enable medical professionals to detect even very small changes in neuronal function-changes that may go undetected otherwise and which will put the individual at a greater risk for secondary complications (65).

Results from fMRI assessments are obtained by measuring changes in local blood oxygen level-dependent (BOLD) contrast levels (65). A prospective fMRI study conducted on eight college football players found that those athletes who experienced an mTBI during the season showed increased BOLD responses while completing memory, sequencing, and sensorimotor tasks. These responses were increased when compared to their own baseline scan at the beginning of the season, but were also increased when compared to the other players with no reports of an mTBI (65). Results from this study imply that the athletes who have sustained an mTBI require more input from neighboring neuronal networks in order to complete the same tasks as controls, probably due to axonal damage near the area of impact (65).

Another study using fMRI and the n-back paradigm (a visual working memory test) demonstrated BOLD signal changes between a preseason baseline assessment and a post-injury or an end of the season assessment. These changes were proportional to the number of head collision events and are possibly linked to alterations in glucose (66) and oxygen (67) metabolism after head impact (65). With these findings, fMRI shows promise as a valuable diagnostic and research tool in the detection, assessment, and tracking of mTBI in athletes.

### Functional Imaging-Magnetoencephalography

Magnetoencephalography (MEG) is another functional neuroimaging modality, one that can identify brain areas involved in language perception, language production, somatosensory perception, and motor performance. Like fMRI, brain activity changes are measured during the performance of a task, and so MEG can be used in mapping the functional organization of the brain. Unlike conventional electroencephalogram (EEG), the surface distributions of the magnetic signals arise mainly from the primary or source currents and are not distorted as they spread to the surface of the head. This allows MEG to provide more accurate spatial localization than EEG (68, 69). Resting state MEG data can assist in establishing normal functional connectivity patterns between different areas of the brain (68). Studies have shown that the neurological symptoms experienced following an mTBI may be a result of alterations in these functional connectivity patterns (68, 69). Healthy neuronal tissues produce resting-state MEG data with frequencies above 8 Hz, while injured tissues produce lower frequencies (70). This low-frequency imaging has shown strong potential as a diagnostic imaging biomarker for mTBI. In one study measuring frequencies of 1–4 Hz, a control group was used to generate a diagnostic threshold that allowed 87% positive detection of mTBI (70). In another study, 55 control subjects and 31 mTBI subjects participated in a MEG assessment that analyzed the proportions of long-range vs. short-range connections within functional connectivity patterns. The study showed that the long-range connections accounted for 60% of all connections in mTBI patients vs. 20% in the control group, and this characteristic could distinguish the mTBI subjects from the control group subjects with 100% accuracy (68). With further research MEG has the potential to diagnosis mTBI, as well as the potential for monitoring recovery.

### Transcranial Magnetic Stimulation

Transcranial Magnetic Stimulation (TMS) is a non-invasive brain stimulation technique that is being increasingly used in studying mTBI. TMS introduces an external, focal magnetic field to brain areas to elicit or disrupt brain activity. Often, TMS is used in conjunction with other neuroimaging methods, such as MRI, and is quickly emerging as an important diagnostic tool in investigating motor and speech and language networks in several neurological disorders. In addition to being non-invasive, TMS can be safely used in adolescents and in the patients with head injury. Using TMS, a study found that in athletes who suffered a concussion, the severity of concussion correlated with degree of inhibition in the motor cortex. This pattern was further exaggerated in athletes who sustained a repeat concussion, indicating to the cumulative effects of repeated injuries (71). In addition, studies of motor excitatory and inhibitory networks using TMS demonstrate persistent functional abnormalities long after symptom recovery and neuropsychological tests return to baseline (72–75). These findings further highlight the need for accurate diagnosis and long term follow up well-beyond symptom recovery in patients with mTBI.

## Discussion

In this review, we examined the pathophysiological cascade and the neuropsychological tests and advanced neuroimaging techniques employed in studying sport related concussion or mTBI in children. A similar examination of traumatic brain injuries resulting from more direct collision was not included in this review since the injury sustained has different biomechanical mechanisms and load distribution. Such injuries are often associated with different clinical symptoms including fracture, hemorrhage, and brain contusions and likely have different metabolic and pathophysiological sequalae. However, this review is confined to sports related concussion as the research on diagnosis, treatment, and the effects of mTBI is increasing, possibly due to greater awareness of concussion in professional sports. There are many areas of progress, yet there is still much to be done. One area that is markedly lacking is the longitudinal follow-up of youth that play sports, and the incidence of long-term post-concussion sequelae. It is unclear whether, and to what extent, concussions sustained in early life increase the risk for other neurological disease processes in later life.

Diagnosis and management of concussion, while much improved over the past 20 years, still is an area that needs further research in the areas of neuropsychological testing and neuroimaging. There remains a lack of evidence speaking to the superiority within and across neuropsychological tests and modalities (e.g., paper and pencil vs. computerized; baseline model vs. norm-referenced). Current recommendations for inclusion of neuropsychological testing in vulnerable populations and in return-to-school decision making is notable; however, general assessment and management should be a multimodal strategy. Wearable accelerometers can be used to assess the threshold of impact needed to initiate an injury and further explore the cumulative effects of subconcussive impacts to the head, particularly in young children and adolescents. Additionally, neuroimaging techniques are crucial to fully understanding the extent of an injury, but advanced techniques such as DTI, fMRI, MEG, and TMS need further development before they can be used clinically to diagnose concussion or determine return-to-play. Extensive research is needed in these areas across all age groups in order to develop more sensitive diagnosis methods, improve clinical guidelines, and prevent avoidable long-term deficits.

## Future Directions

A Recent study reported increased blood levels of T-tau in ice hockey players with sports-related concussion. The concentrations of T-tau were highest immediately after the injury, and the T-tau concentrations at 1 h after concussion predicted the time to recovery (76). Future studies should be directed toward replicating this promising finding in order to significantly improve the way clinicians diagnose and manage sports related concussions and make return-to-play decisions. Toward this end, the NCAA-U.S. Department of Defense Concussion Assessment, Research and Education (CARE) Consortium (http://www.ncaa.org/sport-science-institute/topics/ncaa-dod-care-consortium) was launched in 2014 as the largest study on concussion and repeated head trauma in athletes. Thirty campuses across the country are participating in this study to evaluate both acute and long term effects of head injury in athletes. In particular, the study hopes to define the clinical evolution of concussion and identify the neurobiological underpinning of concussion by using accelerometer telemetry, advanced neuroimaging and biological markers including genetic testing. The long term follow-up phase of the study started in the winter of 2018 and will follow the athletes for 4 years after their athletic career has ended. It is expected that the findings from this study will provide crucial information to better understand the pathophysiology of concussion as well as toward improved diagnosis and management of acute and long term effects of concussion from sport participation. It is also hoped that the neuroimaging, blood and genetic testing can help determine return-to-play. Despite being the largest such study, women athletes continue to be under represented in this consortium. Even as we await the results from this study, large scale studies must also to be initiated in children of both genders playing in a variety of organized sports from a very young age. The advances in neuroimaging hardware and computational capabilities will continue to improve the spatial and temporal resolutions of these techniques and the accuracy of these methods in the diagnosis of sports related concussion will continue to improve in the coming years.

## Author Contributions

All authors take responsibility for the integrity and the accuracy of the review. SN and BB: study concept and design. CC, KC, AS, SN, and BB: literature review. CC, KC, AS, JT, SN, and BB: data analysis and interpretation, drafting and editing of the manuscript, contributing important intellectual content in manuscript review.

### Conflict of Interest Statement

The authors declare that the research was conducted in the absence of any commercial or financial relationships that could be construed as a potential conflict of interest.

## Abbreviations

AAN: American Academy of Neurology
AMS: American Medical Society of Sports Medicine
ANAM: Automated Neurocognitive Assessment Metrics
BOLD: Blood oxygen level-dependent
CNT: Computerized neuropsychological tests
CISG: Concussion in Sports Group
CRI: Concussion Resolution Index
CT: Computed tomography
DTI: Diffusion tensor imaging
EEG: Electroencephalogram
fMRI: Functional MRI
ImPACT: Immediate Post-Concussion Assessment and Cognitive Test
MEG: Magnetoencephalography
MRI: Magnetic resonance imaging
MRS: Magnetic resonance spectroscopy
mTBI: Mild traumatic brain injury
TMS: Transcranial Magnetic Stimulation.
